# Immuno-PET Monitoring of CD8^+^ T Cell Infiltration Post ICOS Agonist Antibody Treatment Alone and in Combination with PD-1 Blocking Antibody Using a ^89^Zr Anti-CD8^+^ Mouse Minibody in EMT6 Syngeneic Tumor Mouse

**DOI:** 10.1007/s11307-022-01781-7

**Published:** 2022-10-20

**Authors:** Hasan Alsaid, Shih-Hsun Cheng, Meixia Bi, Fang Xie, Mary Rambo, Tinamarie Skedzielewski, Bao Hoang, Sunish Mohanan, Debra Comroe, Andrew Gehman, Chih-Yang Hsu, Kamyar Farhangi, Hoang Tran, Valeriia Sherina, Minh Doan, M. Reid Groseclose, Christopher B. Hopson, Sara Brett, Ian A. Wilson, Andrew Nicholls, Marc Ballas, Jeremy D. Waight, Beat M. Jucker

**Affiliations:** 1 Bioimaging, IVIVT, GlaxoSmithKline, Collegeville, PA 19426 USA; 2 Immuno-Oncology Research Unit, GlaxoSmithKline, Collegeville, PA USA; 3 Non-Clinical Safety, IVIVT, GlaxoSmithKline, Collegeville, PA USA; 4 Integrated Biological Platform Sciences, GlaxoSmithKline, Collegeville, PA USA; 5 Research Statistics, GlaxoSmithKline, Collegeville, PA USA; 6 Oncology Cell Therapy Research Unit, GlaxoSmithKline, Hertfordshire, UK; 7 ImaginAb, Inglewood, CA USA; 8 Bioimaging, IVIVT, GlaxoSmithKline, Hertfordshire, UK; 9 Oncology Clinical Development, GlaxoSmithKline, Collegeville, PA USA; 10 Clinical Imaging, GlaxoSmithKline, Collegeville, PA USA

**Keywords:** ICOS agonist, PD-1 blocking, CD8^+^ T cells, ^89^Zr PET imaging, Imaging mass cytometry, Radiomics

## Abstract

**Purpose:**

The presence and functional competence of intratumoral CD8^+^ T cells is often a barometer for successful immunotherapeutic responses in cancer. Despite this understanding and the extensive number of clinical-stage immunotherapies focused on potentiation (co-stimulation) or rescue (checkpoint blockade) of CD8^+^ T cell antitumor activity, dynamic biomarker strategies are often lacking. To help fill this gap, immuno-PET nuclear imaging has emerged as a powerful tool for *in vivo* molecular imaging of antibody targeting. Here, we took advantage of immuno-PET imaging using ^89^Zr-IAB42M1-14, anti-mouse CD8 minibody, to characterize CD8^+^ T-cell tumor infiltration dynamics following ICOS (inducible T-cell co-stimulator) agonist antibody treatment alone and in combination with PD-1 blocking antibody in a model of mammary carcinoma.

Procedures.

Female BALB/c mice with established EMT6 tumors received 10 µg, IP of either IgG control antibodies, ICOS agonist monotherapy, or ICOS/PD-1 combination therapy on days 0, 3, 5, 7, 9, 10, or 14. Imaging was performed at 24 and 48 h post IV dose of ^89^Zr IAB42M1-14. In addition to ^89^Zr-IAB42M1-14 uptake in tumor and tumor-draining lymph node (TDLN), 3D radiomic features were extracted from PET/CT images to identify treatment effects. Imaging mass cytometry (IMC) and immunohistochemistry (IHC) was performed at end of study.

**Results:**

^89^Zr-IAB42M1-14 uptake in the tumor was observed by day 11 and was preceded by an increase in the TDLN as early as day 4. The spatial distribution of ^89^Zr-IAB42M1-14 was more uniform in the drug treated *vs.* control tumors, which had spatially distinct tracer uptake in the periphery relative to the core of the tumor. IMC analysis showed an increased percentage of cytotoxic T cells in the ICOS monotherapy and ICOS/PD-1 combination group compared to IgG controls. Additionally, temporal radiomics analysis demonstrated early predictiveness of imaging features.

**Conclusion:**

To our knowledge, this is the first detailed description of the use of a novel immune-PET imaging technique to assess the kinetics of CD8^+^ T-cell infiltration into tumor and lymphoid tissues following ICOS agonist and PD-1 blocking antibody therapy. By demonstrating the capacity for increased spatial and temporal resolution of CD8^+^ T-cell infiltration across tumors and lymphoid tissues, these observations underscore the widespread potential clinical utility of non-invasive PET imaging for T-cell-based immunotherapy in cancer.

**Supplementary Information:**

The online version contains supplementary material available at 10.1007/s11307-022-01781-7.

## Introduction

Antibody-mediated blocking of the immune checkpoint receptors CTLA-4 and PD-(L)1 has been a mainstay in the battle against cancer; however, despite durable responses across tumor indications, the majority of cancer patients fail to respond to these agents. Alternative therapeutic approaches addressing different immune pathways or enhancing existing activity are needed to broaden and deepen the effects for most patients.

Inducible T-cell co-stimulator (ICOS, CD278) is a co-stimulatory receptor with structural and functional homology to the B7/CD28 immunoglobulin superfamily. ICOS is upregulated on activated CD4^+^ and CD8^+^ T cells after engagement of the T-cell receptor with cognate antigen [1, 2]. Subsequent binding of the ICOS ligand (ICOSL, CD275) to ICOS induces co-stimulatory signaling, promoting general T-cell proliferation, survival, cytokine production, and the cytotoxic function of CD8 + T cells. Combination strategies between co-stimulatory agents and PD-(L)1 or CTLA-4 inhibitors have the potential to improve antitumor responses across different tumor indications [3, 4]. Accordingly, improved antitumor efficacy has been observed with the combination of anti-PD-1 or anti-CTLA-4 in the context of ICOS co-stimulation (i.e., forced ICOSL expression and/or ICOS agonist antibody) in mouse syngeneic tumor models [5–7]. Moreover, upregulation of ICOS on T cells following anti-CTLA-4 checkpoint blocking has also been linked to improved clinical outcomes [8, 9].

Current clinical methods to monitor response to immunotherapy in cancer patients are largely based on ex vivo lymphocyte phenotyping in tumor biopsies and/or whole blood. These methods provide valuable information regarding the treatment effect but are limited by tumor microenvironment heterogeneity, reduced capacity for deep immunokinetic evaluation, limited longitudinal assessment due to feasibility of multiple biopsies, and failure to provide a more systemic view of therapeutic effects. To fill some of these gaps, immuno-PET nuclear imaging has emerged as a powerful tool for *in vivo* molecular imaging [10, 11]. Currently, there are multiple strategies for non-invasive imaging of T cells [12, 13] using a variety of imaging techniques including MRI [14], optical [15], and PET [16–23]. However, most of these techniques are either not clinically translatable, require ex vivo labeling of cells, or are not specific to CD8^+^ effector T cells. ImaginAb, Inc has developed a minibody targeting CD8^+^ T cells [22, 24] to be used as a PET imaging agent after radiolabeling with ^89^Zr to monitor CD8^+^ T-cell infiltration in response to checkpoint inhibitor treatment in tumors and in other lymphoid organs. This minibody is an effector-less 80 kDa antibody fragment with improved diffusion/transport in target tissues and a reduced half-life (*e.g.*, serum, tissue), which allows for repeated dosing and multiple imaging sessions during therapeutic intervention. A phase 1 clinical trial using the humanized CD8^+^ minibody (IAB22M2C) was recently completed for which safety, optimal dosing and imaging time points, pharmacokinetic (PK) and biodistribution properties were assessed [25, 26]. Notably, a phase 2 clinical trial assessing IAB22M2C in solid tumor cancer patients receiving standard of care immunotherapy has since been initiated [27, 28].

To evaluate the utility of this technology in the context of cancer immunotherapy, we used ImaginAb CD8^+^ T-cell (^89^Zr-IAB42M1-14, anti-mouse CD8 minibody) based PET nuclear imaging to selectively monitor CD8^+^ T-cell migration following treatment of EMT6 (mammary carcinoma) tumor-bearing mice with anti-ICOS agonist mAb as a monotherapy and in combination with PD-1 blocking antibody [6]. The PET imaging results were validated using CD8^+^ IHC performed on tumor tissues collected at the terminal timepoint for each treatment cohort.

In support of the *in vivo* PET imaging, additional imaging and image analysis approaches were performed across anatomical, histological, and cellular levels to reveal the distinct CD8^+^ T-cell dynamics following exposure to the different therapeutic antibodies. Investigation into the tumor microenvironment complexity and treatment-associated changes was achieved using imaging mass cytometry (IMC). IMC is a highly multiplexed imaging approach used for deep spatial characterization of tissues at the cellular level, enabling visualization of multiple cell populations in a single image [29]. This level of granularity lends itself to novel analyses like spatial distribution of immune populations and/or markers of interest. In this study, the *in situ* distribution of a panel of 11 protein markers was analyzed in tumor tissues by IMC and an image analysis pipeline was built to elucidate unique cellular populations and compare cell type ratios across treatment groups.

PET radiomics analysis has been used to study the feasibility of using non-invasive image features as a surrogate for cancer phenotypes [30] and to indicate biological abnormalities [31]. In this study, radiomics analysis was employed to fully explore the dynamic process of treatment effect in five organs, including the liver, tumor, draining and non-draining lymph node, and spleen.

## Methods

### EMT6 Syngeneic Tumor Mouse Model

All animal studies were reviewed and approved by the GlaxoSmithKline Institutional Animal Care and Use Committee (IACUC). Six-week-old female BALB/cAnNHsd mice from Envigo (Frederick, MD) were used for these studies.

EMT6 cells (ATCC: CRL-2755) (P6) were thawed from liquid nitrogen and cultured in Waeymouth’s MB 752/1 with 15% FBS. Cells were sub-cultured 3 times over 8 days. Trypsin/EDTA was used to facilitate cell detachment from the culture flask during subculturing.

On day 8, cells were trypsinized, collected, and centrifuged (1200 rpm for 5 min at room temperature RT) to remove the trypsin solution. The cell pellet was resuspended and washed twice in DPBS. Cell number and viability were measured using the Vi-Cell XR Cell Viability Analyzer 2.03 (Beckman Coulter, Brea, CA). A single-cell suspension was prepared in PBS on the ice at 1 × 106 cells/ml, so that a 100 µL injection would deliver 1 × 105 cells per mouse. Tumor volume was measured twice per week, the length and width of each tumor were measured using hand-held calipers and the tumor volume was calculated using the following formula: volume = (length × (width)^2^)/2.

Seven to eight days after tumor implantation in the flank, when tumors reached approximately 50–100 mm^3^, mice were randomized into various treatment groups using Study Director™ software (Version 4.2.1.3, Studylog, San Francisco, CA) based on tumor volume using a stratified sampling method.

### ^89^Zr-IAB42M1-14 Labeling

The ^89^Zr oxalic acid solution (maximum 200 µl, typically 37–185 MBq) was neutralized by 2 M Na_2_CO_3_. ^89^Zr was complexed with Df-IAB42M1-14 at a ratio of 25 µci/μg of minibody in 0.5 M HEPES buffer pH 7.0 at 37 °C for 1 h. ^89^Zr-IAB42M1-14 was then purified with 30 kD vivaspin (Millipore) and radiochemical purity was determined by radio-TLC using a mobile phase of 20 mM citric acid (pH 5.1) and analytical size exclusion chromatography (Superdex 200 10/150 GL, GE Healthcare) with 20 mM HEPES and 150 mM NaCl (pH 7.3) eluted at a flow rate of 0.25 mL/min.

### Experimental Design and PET/CT Imaging

**Fig. 1:**
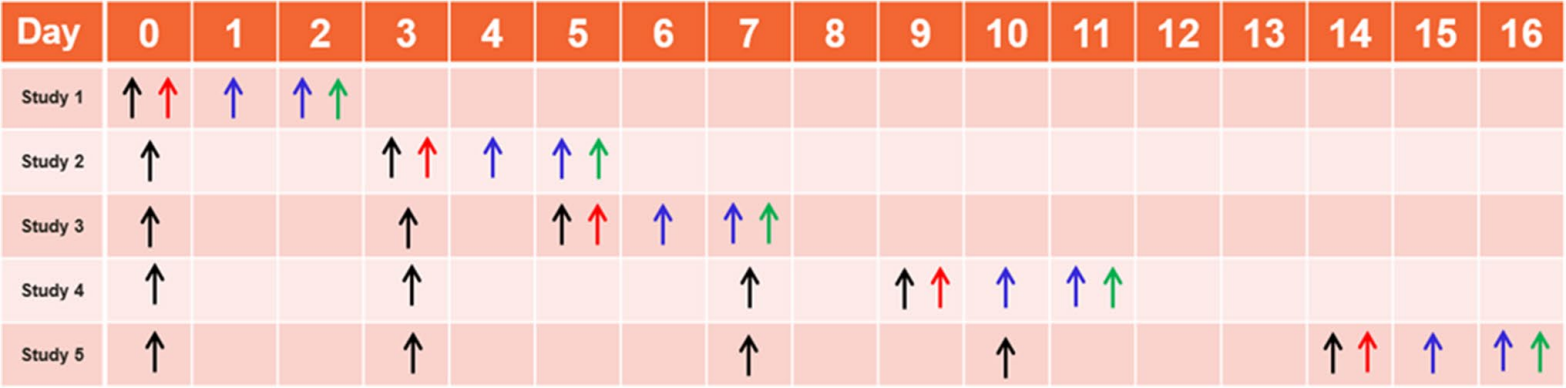
Black arrows represent antibody IP injection, red arrows represent ^89^Zr-CD8^+^ minibody IV injection (^89^Zr-IAB42M1-14 10 µg, 18 µCi/µg), blue arrows represent PET/CT imaging sessions at 24 and 48 h post ^89^Zr-IAB42M1-14 injection, green arrows represent ex vivo IHC

Five different studies were needed to assess the changes in CD8^+^ T-cell infiltration in tumor and tumor-draining lymph node (TDLN) temporally throughout treatment due to the long half-life of ^89^Zr (~ 3.3 days). Mice received a 10 µg IP (100 µl) treatment dose of either IgG control antibodies (Bioxcell, Lebanon, NH), ICOS agonist antibody, monotherapy, (7-ELEVEN.17G9 mouse [m] IgG1, absolute antibody, Oxford, UK), or ICOS agonist antibody (7-ELEVEN.17G9 mouse [m] IgG1) + PD-1 blocking antibody (RMP1-14 rat IgG2a, absolute antibody, and Bioxcell), combination therapy, on days 0, 3, 5, 7, 9, 10, or 14 (Fig. 1). PD-1 blocking antibody was included as a monotherapy in study 5.

*In vivo* PET/CT imaging were performed using a preclinical Inveon system (Inveon Acquisition Workplace v2.1.272, Siemens Medical Solutions USA, Inc) at 24 and 48 h post ^89^Zr-IAB42M1-14 administration (10 μg, 18 μCi/μg on days 0, 3, 5, 9, or 14). Mice were anesthetized using continuously inhaled isoflurane (0.5–2%), and physiological parameters were monitored. A CT scan (80 kV, 500 μA, 4 binning factor) was performed followed by a 10**-**min PET scan (2D FBP with ramp projection filter, matrix size 128 × 128, voxel size of 0.776 × 0.776 × 0.796 mm^3^). 3D regions of interest (ROIs) for the different organs (tumor, tumor-draining lymph node *TDLN*, contralateral non-draining lymph node *NDLN*, spleen, and liver) were defined using Inveon Research Workplace (v4.2, Siemens Medical Solutions USA, Inc.), and quantitative data were presented as a percentage of injected dose per gram of tissue (% ID/g; mean ± SEM). At the terminal timepoint for each treatment cohort, tumor samples were collected for immunohistochemistry assessment (as described in the Supplementary Methods).

The statistical analysis consisted of linear mixed models of tumor volume and *in vivo* T-cell imaging data (performed using SAS 9.4 TS Level 1M5). The cube-root transformation was applied to all tumor volume data, and the day 0 volume was used as a covariate. Additionally, models for both volume and imaging data included treatment group, study day, and their interaction as fixed effects; animal as a random effect; and separate residual variance estimates by study or study day in some cases. From each model, all pairs of treatment groups were compared by their means on the same study day. All tests were two-sided and evaluated at a 0.05 level of significance.

#### Imaging Mass Cytometry (IMC)

Tumor samples collected on day 11 (study 4) were formalin fixed and paraffin embedded, and then sectioned at a 4-µm thickness. The sections were analyzed by imaging mass cytometry on a Hyperion Imaging System (Fluidigm, South San Francisco, CA) after undergoing staining with metal-labeled monoclonal antibodies [29]. Two tumor samples from each treatment group were selected for the analysis. On each tumor section, six to seven representative regions of interest (ROIs) were selected by a pathologist to include tumor parenchyma, stroma, and immune cells. The dimensions for each ROI were approximately 1 mm × 1 mm. Antibodies were obtained in carrier-free buffer and then labeled using the MaxPar antibody conjugation kit (Fluidigm). Antibodies used in this study are listed in Supplementary Data (Table S1). Heat-induced antigen retrieval was optimized to be 95 °C in an EDTA-based pH 9 buffer for 1 h. The Hyperion instrument settings were optimized as previously reported [29], and the imaging pixel diameter was fixed as 1 µm. The CyTOF software version 7 was used for data acquisition.

Imaging mass cytometry data were analyzed using an in-house developed analysis pipeline (as described in the Supplementary Methods).

### Radiomics Feature Analysis

Radiomics features extracted from *in vivo* PET and CT imaging cohorts may help reveal subtle structural changes occurring in the tumor and involved lymph nodes at early timepoints. A total of 4780 radiomics features were extracted from both imaging modalities using two frameworks, CellProfiler [32] and pyradiomics [33]. For each organ, CellProfiler computed 756 features and pyradiomics computed 200 features, including shape, intensity, granularity, and texture. For feature selection, comparisons between six methods were utilized to find treatment related radiomics features, including variance thresholding, univariate feature selection with ANOVA *f*-value and mutual information, logistic regression with L1 penalty, random forest, and AdaBoost. Features from each timepoint were analyzed separately and features chosen by more than four methods were then used for hierarchical clustering.

## Results

### Single-Agent and Combination Therapy Antitumor Efficacy

**Fig. 2:**
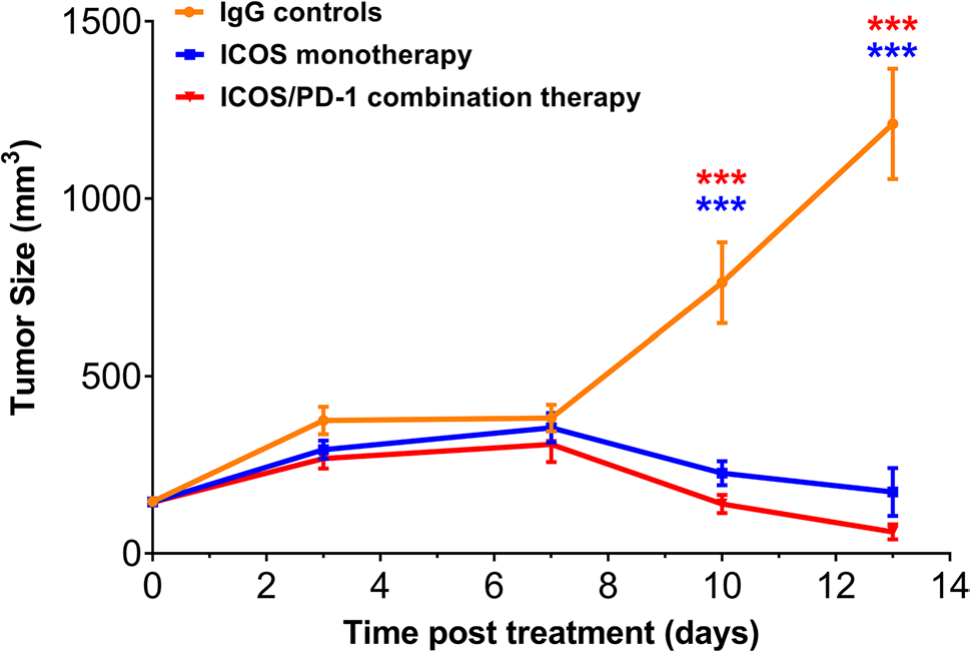
Antitumor activity in a syngeneic murine model of breast cancer (EMT6). The data are representative of study 5 (Fig. 1). Tumor volume was significantly lower on days 10 and 13 post-treatment in both ICOS monotherapy and ICOS/PD-1combination therapy relative to IgG controls group, ***P < 0.001. A linear mixed model with animal as a random effect was used and all pairwise group comparisons were performed within the same day

Antitumor efficacy is shown for study 5 as this study covered the longest treatment period (13 days post-treatment, Fig. 2). Antitumor efficacy is shown for study 5 as this study covered the longest treatment period (13 days post-treatment, Fig. 2)). Following an initial engraftment period, unabated EMT6 tumor growth was observed in the IgG controls group from day 7 through day 13. By contrast, treatment with either ICOS agonist antibody or the combination of ICOS agonist with PD-1 blocking antibodies resulted in a significant and progressive reduction in tumor volume (Fig. 2). Treatment with PD-1 monotherapy showed similar progressive and significant reduction in tumor volume (Figure S1). Notably, by day 15, several mice in the treatment arms had no palpable tumor at time of imaging (*N* = 4 of 12, *N* = 4 of 12, and *N* = 9 of 12 mice in ICOS, PD-1 monotherapies and ICOS/PD-1 combination therapy, respectively).

### *In Vivo* PET/CT Imaging Highlights Treatment-driven Tumor Infiltration of CD8^+^ T Cells

**Fig. 3:**
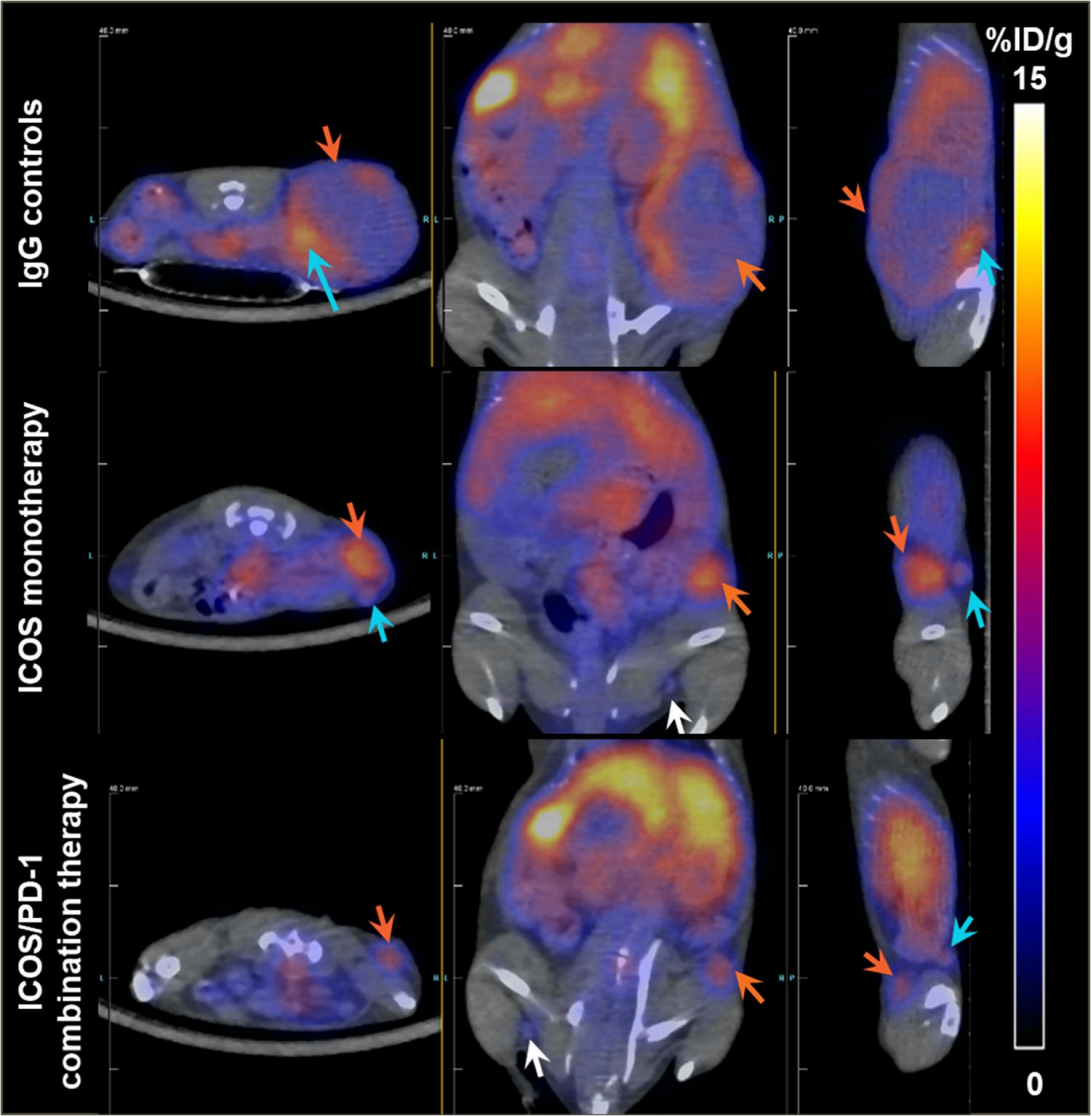
Representative co-registered PET/CT images (study 5 on day 15) are presented in transverse, coronal, and sagittal orientations from left to right. Upper panel: A mouse receiving IgG controls showed heterogenous ^89^Zr– IAB42M1-14 minibody uptake in the tumor with limited uptake in the tumor core. Middle panel: A mouse receiving ICOS agonist monotherapy showed high and homogenous minibody uptake in a small tumor. Lower panel: A mouse receiving ICOS/PD-1 combination therapy showed moderate homogenous minibody uptake in a small tumor and in the TDLN; IHC showed low CD8^+^ T-cell infiltration in the tumor periphery and infiltrating into the tumor parenchyma. Orange arrows indicate tumor, blue arrows indicate TDLN, and white arrow indicates non-draining lymph node

Representative 2D co-registered PET/CT images in three orthogonal orientations obtained on day 15 (study 5) are shown in Fig. 3. Generally speaking, distinct CD8^+^ T-cell distribution patterns were observed between control and therapeutic (single and combination) groups. For example, in an IgG controls–treated mouse, ^89^Zr-IAB42M1-14 minibody uptake was modest and heterogenous in both the tumor and the proximal draining lymph node (Fig. 3, upper panel) with a high uptake in the tumor periphery. Higher ^89^Zr-IAB42M1-14 minibody uptake was observed in the tumor in a mouse responding to ICOS monotherapy (Fig. 3, middle panel). This mouse also demonstrated limited uptake in the proximal draining lymph node. More moderate, but homogenous, ^89^Zr-IAB42M1-14 minibody uptake was observed in a tumor responding to ICOS/PD-1 combination therapy (Fig. 3, bottom panel). Interestingly, moderate uptake was also observed in the proximal draining lymph node.

### Quantitation of Therapy-driven Tumor and Draining Lymph Node Biodistribution for ^89^Zr-IAB42M1-14

**Fig. 4:**
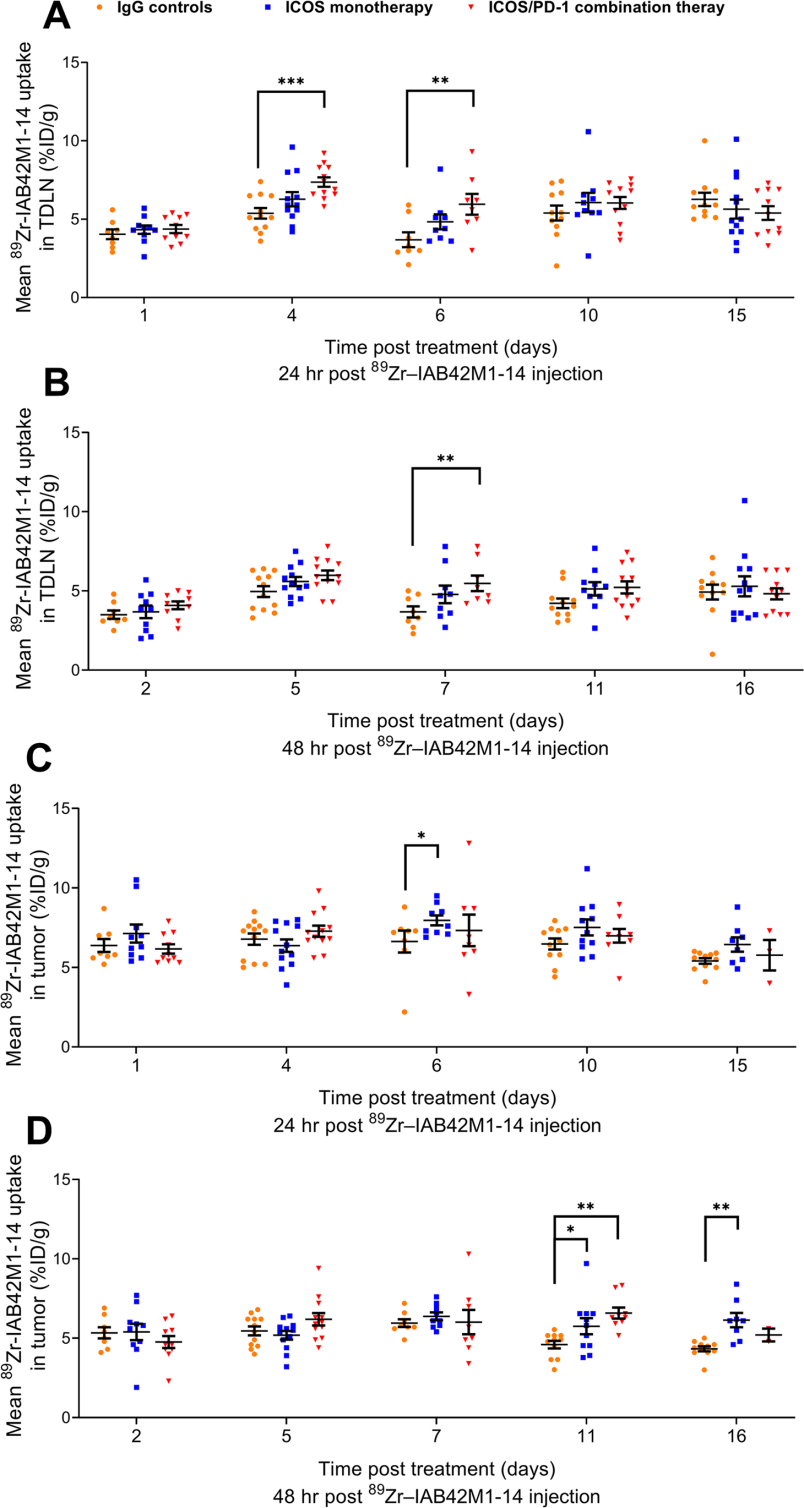
*In vivo* ^89^Zr–IAB42M1-14 minibody uptake in TDLN (**A**, **B**) and in tumor (**C**, **D**) following treatment with ICOS monotherapy or ICOS/PD-1 combination, as measured using PET/CT imaging at 24 h (**A, C**) or 48 h (**B**, **D**) post minibody injection. Minibody uptake in TDLN was significantly higher in the ICOS/PD-1 combination group on days 4, 6, and 7, compared to IgG controls group. The uptake in the tumor was significantly higher in the ICOS monotherapy on days 6, 11, and 16, and in the ICOS/PD-1 combination group on day 11, compared to IgG controls group. *P < 0.05, **P < 0.01, ***P < 0.001. A linear mixed model with animal as a random effect was used and all pairwise group comparisons were performed within the same day

To assess the dynamic of CD8^+^ T cell infiltration in all 5 studies over time, uptake of ^89^Zr-IAB42M1-14 minibody in various organs was quantified via tissue ROI segmentation of PET images (%ID/g). In the proximal draining lymph node (Fig. 4A–B), there was significantly higher uptake in the ICOS/PD-1 combination therapy group on days 4, 6, and 7 relative to the IgG controls group. Notably, uptake of ^89^Zr-IAB42M1-14 minibody in the contralateral non-draining lymph node (Supplementary Data, Figure S2) was significantly higher in treatment groups as compared to the IgG controls group (day 16 for ICOS monotherapy; days 4, 10, and 11 for ICOS/PD-1 combination therapy). ^89^Zr-IAB42M1-14 minibody uptake in tumor (Fig. 4C–D) was significantly higher in treatment groups as compared to the IgG controls group (days 6, 11, and 16 for ICOS monotherapy; day 11 for ICOS/PD-1 combination therapy). Notably, no differences were observed between ICOS monotherapy and ICOS/PD-1 combination therapy in tumor and in the proximal draining lymph node.

Outside of the tumor and lymph nodes, uptake of ^89^Zr-IAB42M1-14 minibody (Supplementary Data) was also significantly higher in peripheral compartments of ICOS and ICOS/PD-1 treated animals relative to IgG controls. Specifically, splenic uptake was significantly higher in the ICOS-treated group on day 16 and days 4 and 16 in the ICOS/PD-1 combination group (Figure S3). Increased liver uptake was also observed for the ICOS/PD-1 combination group on days 4, 5, and 11 relative to the IgG controls group (Figure S4).

In study 5 where PD-1 monotherapy was included, no differences were observed in the proximal draining lymph node between all groups. However, uptake of ^89^Zr-IAB42M1-14 minibody in tumor was significantly higher in PD-1 monotherapy on day 15, and day 16 as compared to the IgG controls group (Figure S5).

### Immunohistochemistry Score of CD8^+^ T Cells in Therapy-driven Tumor

**Fig. 5:**
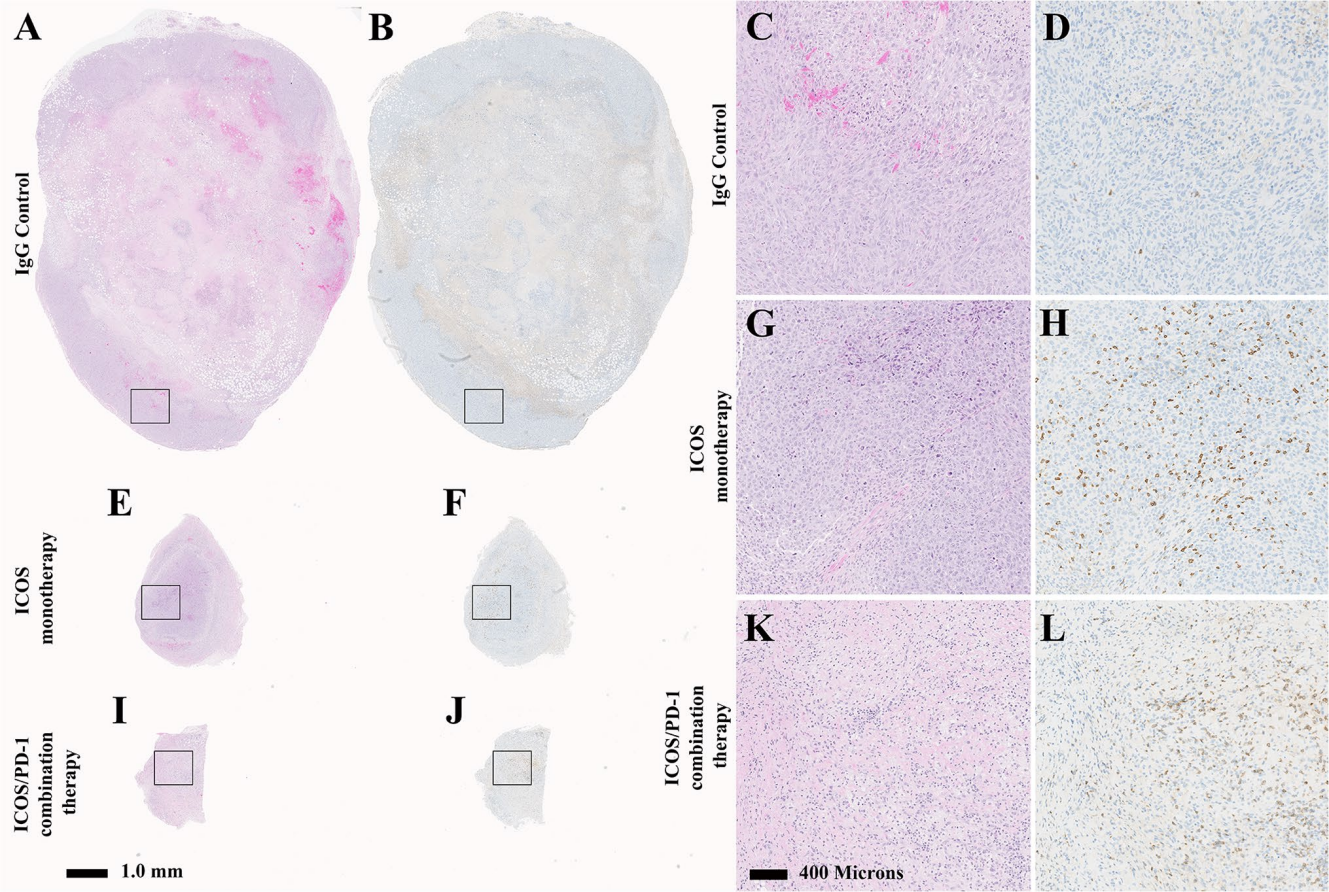
Corresponding H&E (**A**, **E**, **I**, **C**, **J**, **K**) and IHC (**B**, **F**, **J**, **D**, **H**, **L**) staining of the PET/CT images presented in Fig. 3. Discernable differences in tumor size between the IgG controls group (**A**), ICOS monotherapy (**E**), and ICOS/PD-1 (**I**) combination therapy. Higher magnification photomicrographs showing discernable differences between viable tumor cells: necrosis ratio between the IgG controls groups (**C**), ICOS monotherapy (**G**), and ICOS/PD-1 combination therapy (**K**). IHC showed low CD8^+^ T-cell infiltration in the parenchyma in the IgG controls group (**D**), high CD8^ +^ T-cell infiltration in the tumor in the ICOS monotherapy (**H**), low CD8^ +^ T-cell infiltration in the tumor periphery and infiltrating into the tumor parenchyma in the ICOS/PD-1 combination therapy (**L**)

IHC for CD8^+^ T cells was performed in order to confirm the *in vivo* PET imaging results obtained using the ^89^Zr-IAB42M1-14 minibody (Fig. 5). IHC reflected low CD8^+^ T-cell infiltration in the core of the tumor in the IgG controls group on days 5, 7, 11, and 16 (Table 1). In these larger tumors, some CD8^+^ T-cell infiltration was observed in the periphery consistent with the *in vivo* images in these tumors. In contrast, ICOS monotherapy and ICOS/PD-1 combination group exhibited higher and more homogeneously-distributed CD8^+^ T-cell tumor infiltration as reflected by a higher IHC scoring in the core relative to the IgG controls (Table1).

**Table 1:** Pathology score of tumor CD8^+^ T-cell infiltration

Study number (day)	Group	Periphery	Core	Overall
Study 2 (day 5)	IgG controls	1 (1–2)	1 (1–1)	1 (1–2)
	ICOS	2 (1–2)	1 (1–2)	1 (1–2)
	ICOS/PD-1	2 (1–3)	2 (1–3)	2 (1–3)
Study 3 (day 7)	IgG control	1.5 (1–2)	1 (0–2)	1.5 (1–3)
	ICOS	2 (1–3)	1 (0–3)	2 (1–3)
	ICOS/PD-1	2 (1–2)	2 (0–3)	2 (1–3)
Study 4 (day 11)	IgG control	1 (0–2)	1 (0–2)	1 (0–2)
	ICOS	1 (1–2)	2 (0–3)	1.5 (1–3)
	ICOS + PD-1	1 (1–2)	2 (1–3)	2 (1–2)
Study 5 (day 16)	IgG control	1 (1–1)	1 (1–1)	1 (1–1)
	ICOS	1 (1–1.5)	2 (1–2)	2 (1–2)
	ICOS + PD-1	1 (1–1)	1.5 (1–2)	1.5 (1–2)

Summarized IHC score for IgG controls, ICOS agonist monotherapy, and ICOS/PD-1 combination therapy at days 5, 7, 11, and 16 for tumor periphery, core, and overall. Data is presented as group median score (minimum score – maximum score)

### Imaging Mass Cytometry of Cell Populations in Therapy-driven Tumor

Imaging mass cytometry (IMC) biomarker profiling was conducted on ex vivo tumor tissue samples. An image analysis pipeline including cell type calling, nearest neighbor, and proximity analyses was developed to unveil the spatial cell distribution and infiltration landscapes.

**Fig. 6:**
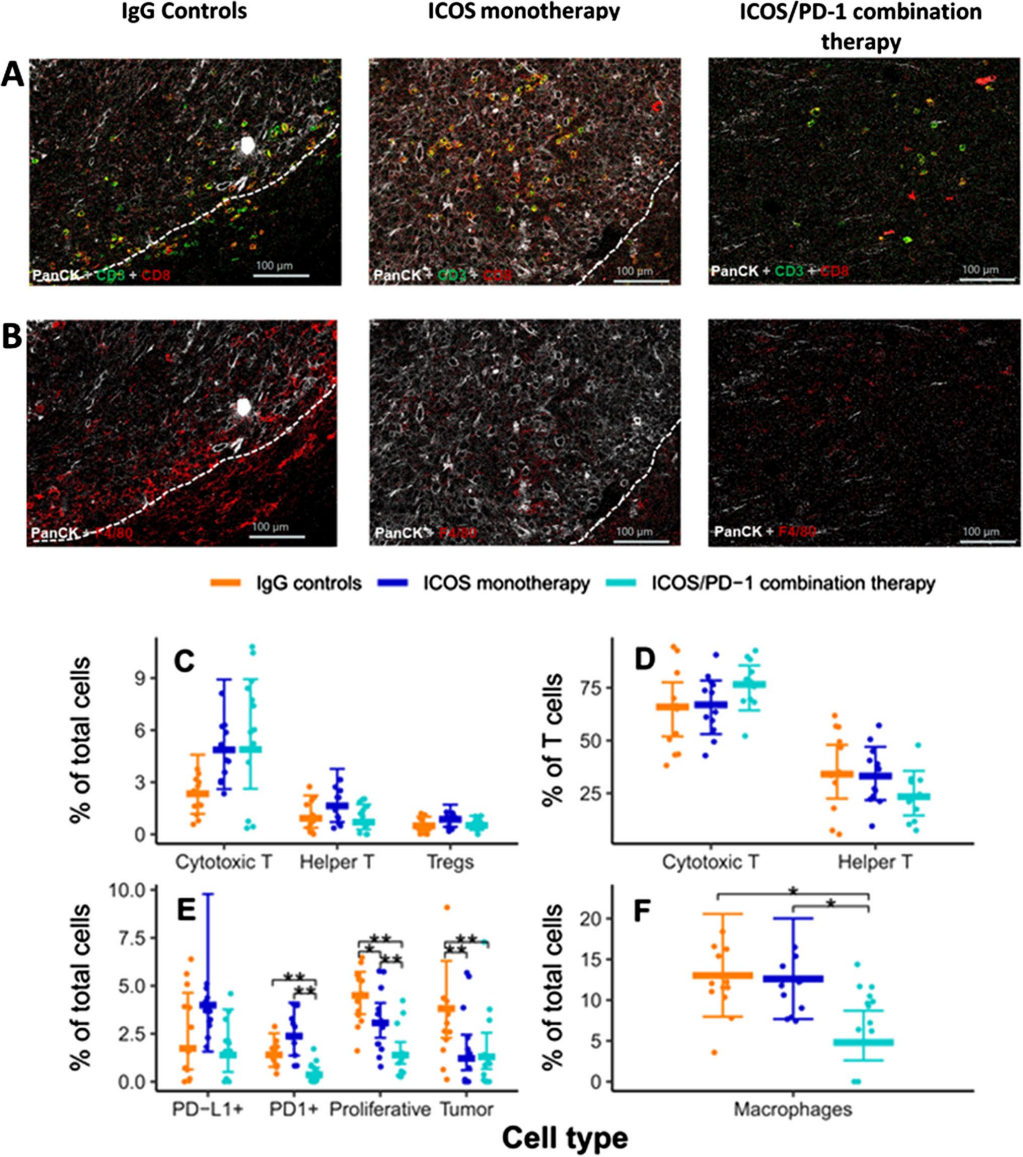
Representative mass cytometry images are shown in panels **A** and **B**. The dotted lines represent the approximate border of the tumors. **A** Pan cytokeratin^+^ (PanCK; white, tumor marker) and CD3^+^ CD8^+^ (green + red = yellow, cytotoxic T cells). In the IgG controls–treated sample, cytotoxic T cells were observed in the peripheral region of the tumor; in the ICOS monotherapy sample, cytotoxic T cells infiltrated into the tumor; in the ICOS/PD-1 combination therapy sample, the tumor was resolved with spreading PanCK^+^ cells accompanied by a few cytotoxic T cells. **B** PanCK^+^ (white, tumor marker) and F4/80^+^ (red, macrophages). In the IgG controls–treated sample, high tumor–macrophage interaction was prevalent in the tumor; in the ICOS monotherapy sample, tumor cells showed higher interaction with cytotoxic T cells when compared with macrophages, whereas macrophages were found mainly in the peripheral region; in the ICOS/PD-1 combination therapy sample, PanCK.^+^ tumor cells were low in number and the proliferative cells were admixed with macrophages and cytotoxic T cells. **C** Scatter plots of the ratio of cytotoxic T cells, helper T cells, and regulatory T cells in the total cell population; **D** scatter plots of the ratio of cytotoxic T cells and helper T cells in the T cell population; and **E**–**F** scatter plots of cell type ratios by cell type with the estimated mean and the 95% confidence intervals for IgG, ICOS monotherapy, and ICOS/PD-1 combination therapy. *P < 0.05, **P < 0.01 (beta regression with animal as a random effect)

Figure 6A shows the spatial relationship between cytotoxic T cells, which is defined as CD3^+^ CD8^+^, and tumor cells (PanCK^+^) on day 11 in a selected ROI from one animal of each group. From the images, it appears that cytotoxic T cells are localized in the peripheral region of the tumor in the IgG controls group. By contrast, cytotoxic T cells infiltrated into the tumor in the ICOS monotherapy and ICOS/PD-1 combination group. Notably, in the ICOS/PD-1 combination group, the tumor was largely resolved, with scattered PanCK^+^ cells accompanied by a few cytotoxic T cells.

Figure 6B illustrates the spatial relationship of macrophages (F4/80^+^) and tumor cells. In the IgG controls group, a high tumor–macrophage interaction was observed. By contrast, macrophages were primarily found in the peripheral region of tumor in the ICOS monotherapy group. In the ICOS/PD-1 combination group, PanCK^+^ tumor cells were low in number and amalgamated with dispersed macrophages. The ratio of cytotoxic T cells and macrophages to total cells are shown in Fig. 6C and F. Other cellular populations included in the image analysis were helper T cells (CD3^+^ CD4^+^), regulatory T cells (Treg, CD4^+^FoxP3^+^), proliferative cells (Ki67^+^) for all cell populations, PD1^+^ cells, and PD-L1^+^ cells. The comparison of the ratio of each cellular population among the three groups is displayed in Fig. 6C and Fig. 6E. The ratio of CD4^+^ over CD3^+^ and the ratio of CD8^+^ over CD3^+^ is shown in Fig. 6D.

### Radiomics Analysis Reveals Early Detection of Treatment Effects

**Fig. 7:**
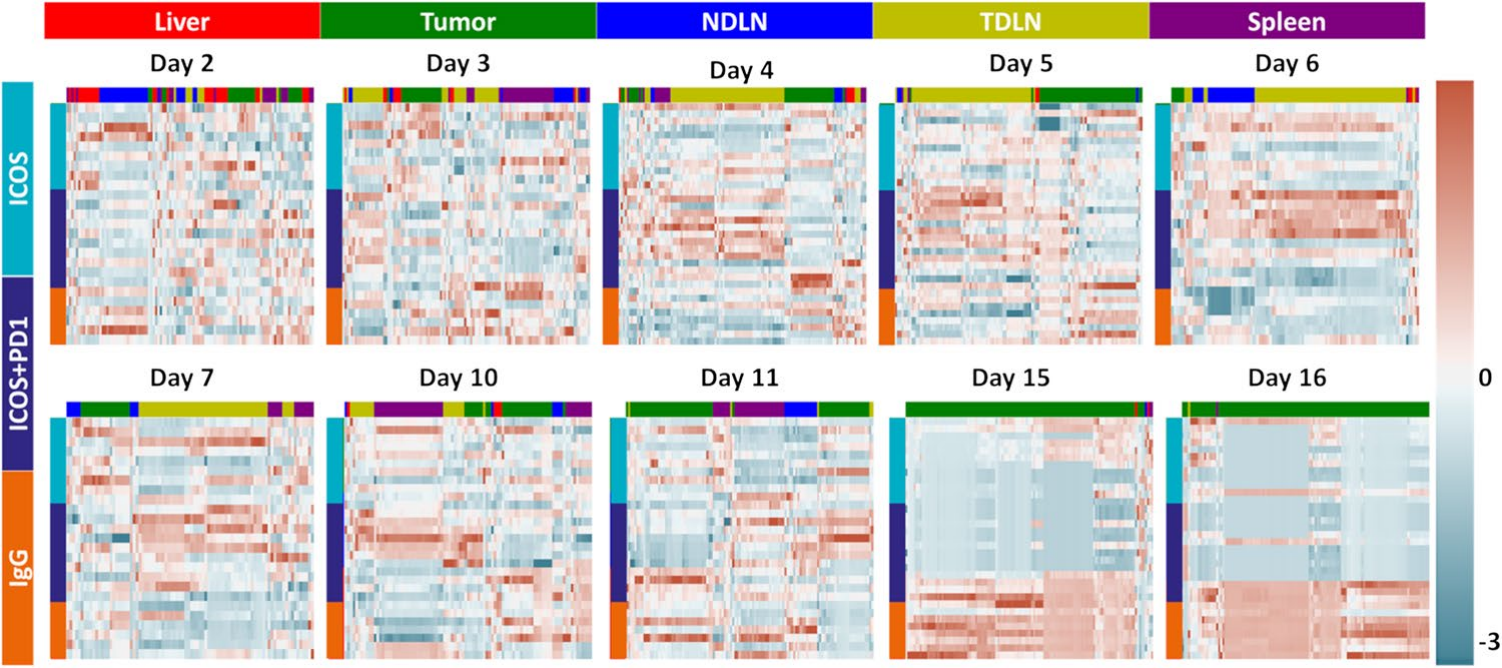
Cluster heatmap for IgG controls, ICOS agonist monotherapy, ICOS/PD-1 combination therapy. The combination group starts expressing different signatures compared to IgG controls as early as day 4. Features from tumor-draining lymph node TDLN (yellow) were highly predictable in earlier timepoints (days 4, 5, 6, 7) while tumor in later timepoints (days 11, 15, 16)

Radiomics translates medical images into quantitative data via handcrafted image features. Combined with data science, biology-related image features are discovered to yield better understanding of the underlying mechanisms. In order to concatenate all the collected data, and potentially extract critical treatment-driven patterns, we employed radiomics-based analyses. Figure 7 shows the clustering heatmap with rows as animals and columns as features. To effectively track temporal change of radiomics profile, the rows were grouped with respect to treatment group (red: IgG controls, green: ICOS monotherapy, blue: ICOS/PD-1 combination therapy). The columns at each date are the selected features with the top color bar depicting the origin organ (red: liver, green: tumor, blue: non-draining lymph node, yellow: tumor-draining lymph node, purple: spleen). The heatmap is expressed in red-blue palette with red indicating high value and blue as low value.

Importantly, feature clustering can be seen as early as day 4, where an inverse in signal was observed between IgG controls and therapeutic groups. Specifically, TDLN exhibited a high signal in the combination group and low overall signals in the IgG controls. Conversely, tumors expressed high signals in the IgG controls group and low signals in treated groups. The expression difference continued throughout the experiment with signals coming from different organs. Starting from day 10, signals from spleen started to show difference with more features coming from tumor in day 11. In days 15 and 16, tumor features were dominantly chosen as predictive features.

## Discussion

In this study, *in vivo* immuno-PET was used to non-invasively assess the kinetics of CD8^+^ T-cell biodistribution and tumor infiltration following ICOS agonist antibody treatment as monotherapy or in combination with PD-1 blocking antibody in the EMT6 murine syngeneic tumor model. This technique allowed us to observe a progressive shift from early heterogeneity in the distribution of tumor-infiltrating CD8^+^ T cells to a more uniform distribution (with greater frequency) following mono/combo immunotherapy. Coupled with imaging mass cytometry, we were able to establish a robust set of tumor- and system-based features to tease apart kinetic pharmacodynamic patterns following cancer immunotherapy.

The ability to monitor the whole-body distribution of T cells in a kinetic fashion enables a greater understanding of biodistribution kinetics, including the potential to observe early uptake of CD8^+^ T cells in the tumor-draining lymph node. For example, while ICOS monotherapy and ICOS/PD-1 combination therapy exhibited a similar effect on CD8^+^ T-cell kinetics in tumors (days 11, 15, and 16 post-treatment), combination therapy resulted in a significantly earlier presence of CD8^+^ T cells in the tumor-draining lymph node (days 4, 6, and 7 post-treatment) relative to the monotherapy. This effect could easily have been missed using alternative monitoring approaches. The presence of CD8^+^ T cells in the draining lymph node prior to appreciable increases in the tumor tissue is not surprising given the role of lymphoid organs in T-cell priming [34]. Notably, others have made similar PET-based observations with respect to the draining lymph node *versus* tumor T-cell kinetics following immunotherapy [18]. These previous findings and our results suggest that local lymph node priming of T cells may be assessed early on after immunotherapy to use as a surrogate for T-cell activation, subsequent infiltration into tumor and therapeutic response. In addition, the whole-body nature of this approach allows one to monitor site-specific *versus* system immune activation. To this notion, we observed a significant increase in ^89^Zr-IAB42M1-14 minibody uptake in the liver and lymphatic system including the non-draining lymph node, and spleen in both ICOS monotherapy and ICOS/PD-1 combination therapy, suggestive of systemic immune activation.

Establishing correlations between tumor T-cell infiltration and reduction in tumor burden proved to be challenging, as only a limited number of tumors demonstrated a graded response or, in the case of ICOS/PD-1 combination therapy, resulted in small to no tumor detection following weeks of treatment. Even with these challenges, we observed clear trends that orthogonal readouts helped to clarify. Indeed, histopathology and imaging mass cytometry confirmed the imaging findings with regard to spatial heterogeneity of PET signal. The larger tumors associated with the IgG controls treatment group showed little to no necrosis, so viability of tissue in the tumor core should not have altered ^89^Zr-IAB42M1-14 minibody uptake and distribution. Indeed, the responding tumors had more homogeneous uptake and they also appeared to be temporally responsive. Similar findings were observed using T-cell targeting ^89^Zr-labeled PEGylated single-domain antibody fragments (VHHs) specific for CD8^+^ to track the presence of intratumoral CD8^+^ T cells in response to CTLA-4 therapy in B16 melanoma tumor-bearing mice [19]. In this study, animals that responded to CTLA-4 therapy showed a homogeneous distribution of the anti-CD8^+^ PET signal throughout the tumor, whereas more heterogeneous infiltration of CD8^+^ T cells correlated with faster tumor growth and worse response. Mouse syngeneic tumor models are widely used as a translatable model to assess novel immune therapies. While tumor heterogeneity with respect to T cell and other immune cell populations can vary significantly even in these models, there is evidence that the absolute T-cell numbers generally correlates with survival [35]. EMT6 mouse tumors inherently express high levels of chemokines, MHC-I, and antigen presentation components [36]. In this model, it was shown that immune cell infiltrates generally decreased with increasing size of tumor and are confined to the invasive margin [36]. These preclinical findings are consistent with immunohistological analysis of human biopsy specimens taken from patients receiving immunotherapy, where nonresponding lesions show a peripheral distribution of CD8^+^ T cells and responders show clear signs of CD8^+^ T cells that penetrate the tumor [37–39].

In addition to supporting some of basic observations from PET analysis, imaging mass cytometry provided a unique opportunity to evaluate the treatment effects on the remodeling of the tumor microenvironment. For example, ICOS monotherapy and ICOS/PD-1 combination therapy significantly decreased the percentage proliferative cells and percentage tumor cells, confirming the efficacy of the treatment. As for immune cells, although not statistically significant due to the large variation among and limited sample size, the percentage of cytotoxic T cells (of total cells) in the ICOS monotherapy and ICOS/PD-1 combination group were approximately double of the IgG controls group. Moreover, the percentage of CD4^+^ non-Treg cells (effector T cells) appeared to increase slightly in the ICOS monotherapy group, whereas there were no clear differences between groups in the percentage CD4^+^ Treg cells. Interestingly, selective analysis of the tumor-infiltrating T cells (of CD3^+^ cells instead of total cells) indicated that the ICOS/PD-1 combination therapy increased the percentage of cytotoxic CD8^+^ T cells and, at the same time, reduced the percentage of CD4^+^ effector cells. Outside of T cells, the percentage of macrophages in the ICOS/PD-1 combination group was significantly lower relative to ICOS monotherapy or IgG controls, an effect that could possibly be attributed to reduced tumor burden with the combination. Finally, ICOS monotherapy increased the percentage PD-(L)1 positive cells relative to IgG controls, an effect that was lost with the addition of PD-1 blocking antibody.

Radiomics complements traditional image analysis by extracting additional image features that may be used to identify potential biologically relevant image patterns. Being hypothesis-free by default, radiomics feature extraction methods, such as those used in this article, yield a large set of parameters to select from, and eventually enable a robust characterization of disease states through morphological fingerprints. For example, radiomics analysis has been used to enable early detection of cancer [40]. When applied to longitudinal studies similar to the presented work, temporal changes in radiomics add significant complementary values in predicting and monitoring disease progression and survival outcome [41]. In this study, radiomics features in the TDLN were significantly different between the IgG controls and therapeutic groups as early as day 4. It demonstrated promising early detection of treatment efficacy by applying data mining to image analysis which is validated by *in vivo* results with statistical significance in TDLN at days 4 and 6. A future development would be using automated segmentation to increase and expand the analysis to more regions of interest. As well as image level data mining for hypothesis-free analysis and explore radiomics in unexpected regions to better understand treatment effect in the entire subject. The biggest limitation in using data mining to evaluate treatment effects is the sample size. In this study, feature selection was conducted using treatment groups as supervised labels and unsupervised clustering for visualizing the feature differences. With increased statistical power, classification models can be built and robustly validated as well as enabling time-dependent analysis such as delta radiomics.

## Conclusion

In summary, using ^89^Zr-IAB42M1-14 minibody PET imaging tracer, CD8^+^ T-cell recruitment following ICOS monotherapy and ICOS/PD-1 combination therapy was kinetically assessed in an EMT6 syngeneic mouse tumor model. ICOS monotherapy and ICOS/PD-1 combination therapy exhibited a similar effect on CD8^+^ T-cell kinetics in tumors; however, an early significant increase of the CD8^+^ minibody uptake in the tumor-draining lymph node was observed following ICOS/PD-1 combination therapy with a less distinct increase following ICOS monotherapy. These data were both confirmed and supplemented with orthogonal techniques that helped feed into a radiomics-based evaluation of immunotherapy-induced tumor and systemic changes. A limitation of this study is that the effect of PD-1 monotherapy on CD8^+^ T-cell kinetics was not fully assessed. In the future, this non-invasive imaging strategy may be used to evaluate ICOS agonist monotherapy or in combination with other immunotherapy and to assess the changes in CD8^+^ T-cell infiltration in tumor throughout the treatment regime. Additionally, it may provide us with a useful tool to understand the relationship between CD8^+^ T-cell flux in tumor and in proximal draining lymph node following immunotherapy. As such, these translational imaging strategies may help in the optimization of early surrogate biomarkers for immunotherapy response in tumors.

## Supplementary Information

Below is the link to the electronic supplementary material.

Supplementary file1 (DOCX 453 KB)
