# Supplementary material for: Immuno-PET Monitoring of CD8+ T Cell Infiltration Post ICOS Agonist Antibody Treatment Alone and in Combination with PD-1 Blocking Antibody Using a 89Zr Anti-CD8+ Mouse Minibody in EMT6 Syngeneic Tumor Mouse
Source: Mol Imaging Biol. 2022 Oct 20;25(3):528–40. doi: 10.1007/s11307-022-01781-7 (PMC10172244; doi:10.1007/s11307-022-01781-7)
Supplement: Supplementary file 1 — Supplementary file1 (DOCX 453 KB) [file 11307_2022_1781_MOESM1_ESM.docx]

**Electronic Supplementary Material**

**Immuno-PET monitoring of CD8^+^ T cell infiltration post ICOS agonist antibody treatment alone and in combination with PD-1 blocking antibody using a ^89^Zr anti-CD8^+^ mouse minibody in EMT6 syngeneic tumor mouse**

Hasan Alsaid^1^, Shih-Hsun Cheng^1^, Meixia Bi^2^, Fang Xie^1^, Mary Rambo^1^, Tinamarie Skedzielewski^1^, Bao Hoang^1^, Sunish Mohanan^3^, Debra Comroe^4^, Andrew Gehman^5^, Chih-Yang Hsu^1^, Kamyar Farhangi^1^, Hoang Tran^5^, Valeriia Sherina^5^, Minh Doan^1^, Reid Groseclose^1^, Christopher B Hopson^2^, Sara Brett^6^, Ian A Wilson^7^, Andrew Nicholls^8^, Marc Ballas^9^, Jeremy D Waight^2^, and Beat M Jucker^10^

^1^Bioimaging, IVIVT, GlaxoSmithKline, Collegeville, PA, USA

^2^Immuno-Oncology Research Unit, GlaxoSmithKline, Collegeville, PA, USA

^3^Non-Clinical safety, IVIVT, GlaxoSmithKline, Collegeville, PA, USA

^4^Integrated Biological Platform Sciences, GlaxoSmithKline, Collegeville, PA, USA

^5^Research Statistics; GlaxoSmithKline, Collegeville, PA, USA

^6^Oncology Cell Therapy Research Unit, GlaxoSmithKline, Hertfordshire, UK

^7^ImaginAb, Inglewood, CA, USA

^8^Bioimaging, IVIVT, GlaxoSmithKline, Hertfordshire, UK

^9^Oncology Clinical Development, GlaxoSmithKline, Collegeville, PA, USA

^10^Clinical Imaging, GlaxoSmithKline, Collegeville, PA, USA

**Corresponding author:**

Hasan Alsaid, Ph.D.

1250 S. Collegeville Rd, Collegeville, PA 19426

Tel. 610-917-6046

Hasan.5.alsaid@gsk.com

**Supplementary Materials**

**Immunohistochemistry**

Tumor Immunohistochemistry (IHC) was performed on a Ventana Discovery Ultra system (Ventana/Roche). Each tumor provided 1-3 spatially distinct tissue sections that were collected, deparaffinized, and hydrated on the Discovery Ultra. Antigen retrieval was performed using Tris based (EDTA) buffer solution, CC1 (Ventana). Primary antibody rat anti-mouse CD8 (1:100 dilution, bioscience) or rat IgG isotype control (2 µg/ml, Invitrogen) were added to the sections for 1 hr followed by secondary incubation with polymer based OmniMap anti-rat HRP (Ventana) and visualized by incubation with ChromaMap DAB detection kit (Ventana). Tissue sections were counterstained with hematoxylin (Ventana) and serial sections were stained with H&E on a Leica autostainer. Whole slide scans (WSS) were obtained using a Nanozoomer slide scanner (Hamamatsu, Bridgewater, NJ), and whole section morphological regions were annotated using HALO (Adobe Systems, San Jose, CA). Areas of necrosis and hemorrhage were excluded from analysis.

For pathology scoring, tumor edge (300 μm from edge of tumor section) and tumor core (entire tumor parenchyma excluding the edge) were delineated using HALO annotation. Semi quantitative analysis of CD8^+^ T cell infiltration was performed by a pathologist scoring and by digital image analysis. A scale of 0-3 was used by the pathologist to represent the degree of CD8^+^ T cell infiltration [0 = No or minimal infiltrate consisting of less than 5% of tumor area, 1 = Low 5-20% of tumor area, 2 = Medium 20-50% of tumor area, 3 = High 50-100% of tumor area].
Semi-quantitative CD8^+^ IHC score data was summarized by the median and range for each treatment group, separately for each tumor region and study day (no statistical testing was performed). In cases where multiple tumor sections were scored for the same region of the same animal, the median score was used for this summary.

**Imaging Mass Cytometry**

Imaging Mass Cytometry data were analysed using an in-house developed analysis pipeline. Briefly, cell segmentation was first performed on the images using machine learning-based pixel classification via Ilastik [1], and propagation of the nuclei pixels to membrane pixels followed by generation of the cell segmentation mask using CellProfiler [2]. Cell segmentation mask was then used towards reading single-cell level average pixel intensities as surrogates for protein expression. Single cell expression data were then z-normalized and fitted by a 2-component Gaussian Mixture model to automatically recognize positively expressing from none-expressing cells at every single channel. Normalized and cleaned single cell protein expression data were then went through a K-means clustering algorithm classifying cells into cell clusters with unique protein expression footprint. Each protein expression footprint was then visualized and assigned to a cell type/phenotype name based on the expression of their canonical markers. Cell type ratios were then calculated for each animal by aggregating the single cell information of all the imaged regions of interest (ROIs) and calculating the ratio of every single recognized cell type/phenotype with respect to the overall cell population in the corresponding sample.

To estimate the differences between treatments, the cell type ratios were modelled using a beta regression with logit link. Any ratios equal to zero were accommodated by adding a pseudo count of ${10}^{-5}$. The p-values were unadjusted because the comparisons of interest were prespecified prior to the data collection process. Due to the logit link, all comparisons between treatment groups are in terms of odds ratios. However, because cell type ratios were small, the odds ratios are approximately equal to the relative risk, i.e., fold changes of cell type ratios between treatment groups. Thus, comparisons of cell type ratios between treatment groups were reported as approximate fold changes. The statistical analysis was performed in statistical software R, version 4.0.2 [3], specifically the package “glmmTMB [4] was used for modelling, the package “emmeans” [5] was used for post-hoc comparisons and p-value calculations.

1. Berg S, Kutra D, Kroeger T, et al. (2019) ilastik: interactive machine learning for (bio)image analysis. Nat Methods 16:1226-1232.

2. McQuin C, Goodman A, Chernyshev V, et al. (2018) CellProfiler 3.0: Next-generation image processing for biology. PLoS Biol 16:e2005970.

3. Team RC (2020) R: A language and environment for statistical computing. R Foundation for Statistical Computing, Vienna, Austria. URL <https://www.R-project.org/>.

4. Brooks ME, Kristensen K, van Benthem KJ, et al. (2017) glmmTMB Balances Speed and Flexibility Among Packages for Zero-inflated Generalized Linear Mixed Modeling. The R Journal 9:378-400.

5. Lenth R (2020) emmeans: Estimated Marginal Means, aka Least-Squares Means. R package version 1.4.8. <https://github.com/rvlenth/emmeans>.

**Table S1.** *IMC Mouse Marker Panel*

| Labeling Metal | Marker | Antibody Clone |
| --- | --- | --- |
| 148Nd | Pan-Keratin | KRT1877R |
| 150Nd | PD-L1 | D5V3B |
| 152Sm | CD8a | EPR20305 |
| 154Sm | CD11b | EPR1344 |
| 156Gd | CD4 | EPR19514 |
| 159Tb | PD-1 | EPR20665 |
| 164Dy | F4/80 | SP115 |
| 165Ho | FoxP3 | EPR22102-37 |
| 166Er | NCR1 | EPR23097-35 |
| 170Er | CD3G | EPR4517 |
| 171Yb | Ki67 | SP6 |
|  |  |  |

**
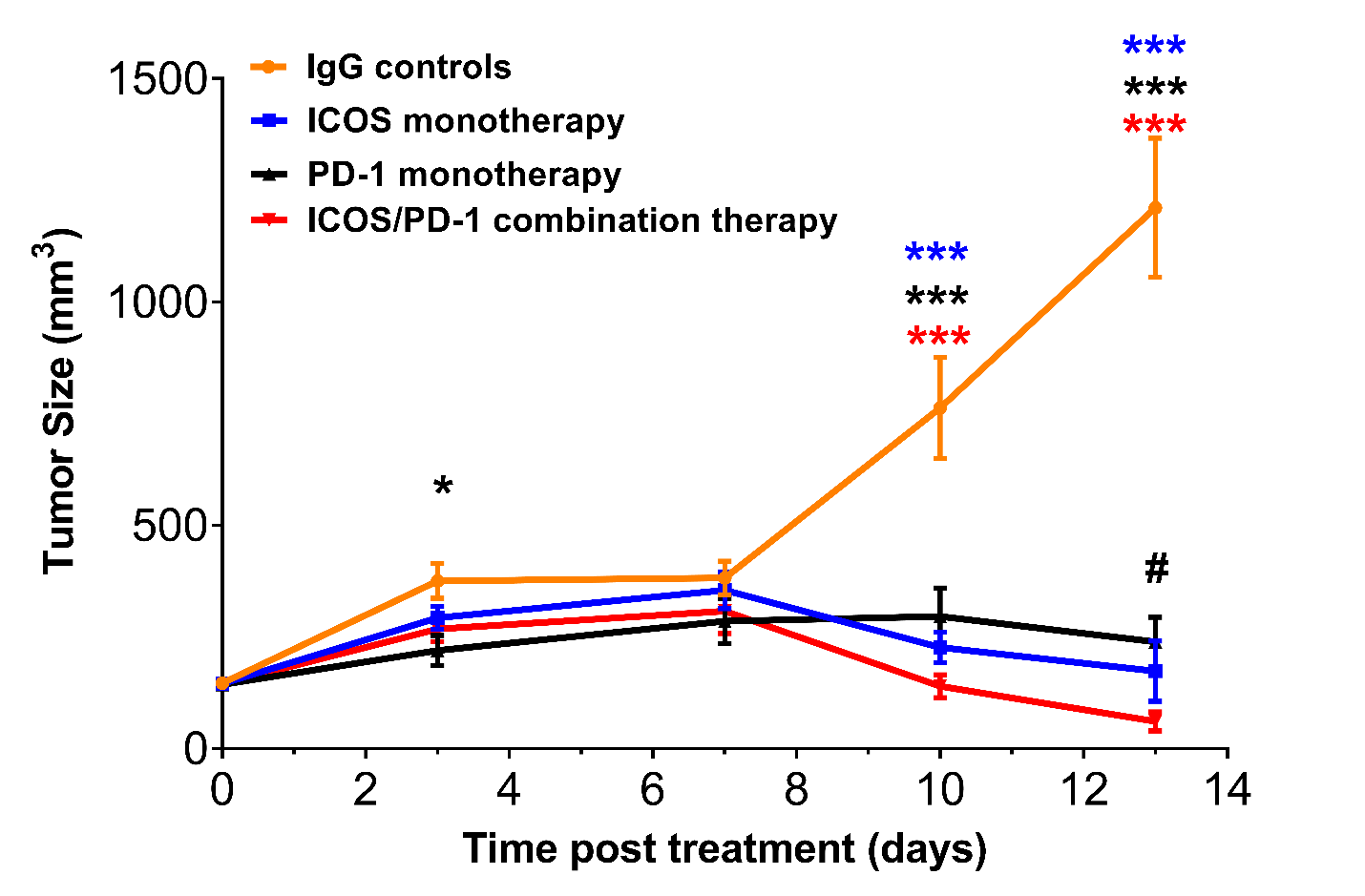
**

**Figure S1.** Antitumor activity in a syngeneic murine model of breast cancer (EMT6). The data are representative of Study 5 (Fig. 1) including PD-1monotherapy. Tumor volume was significantly lower on day 3 post-treatment in PD-1 monotherapy (*P<0.05), and on days 10 and 13 post-treatment in ICOS, and PD-1 monotherapies and ICOS/PD-1combination therapy (***P<0.001) relative to IgG controls group. On day 13 post treatment tumor volume was significantly higher in the PD-1 monotherapy compared to ICOS/PD-1combination therapy (^#^P<0.05). Analysis was performed using a linear mixed model with animal as a random effect, and all pairwise group comparisons within the day. Note that this analysis was conducted in addition to the primary analysis displayed in Fig. 2. that involved only three treatment groups; as a result, this analysis should be considered exploratory, and its findings of statistical significance should be interpreted accordingly.


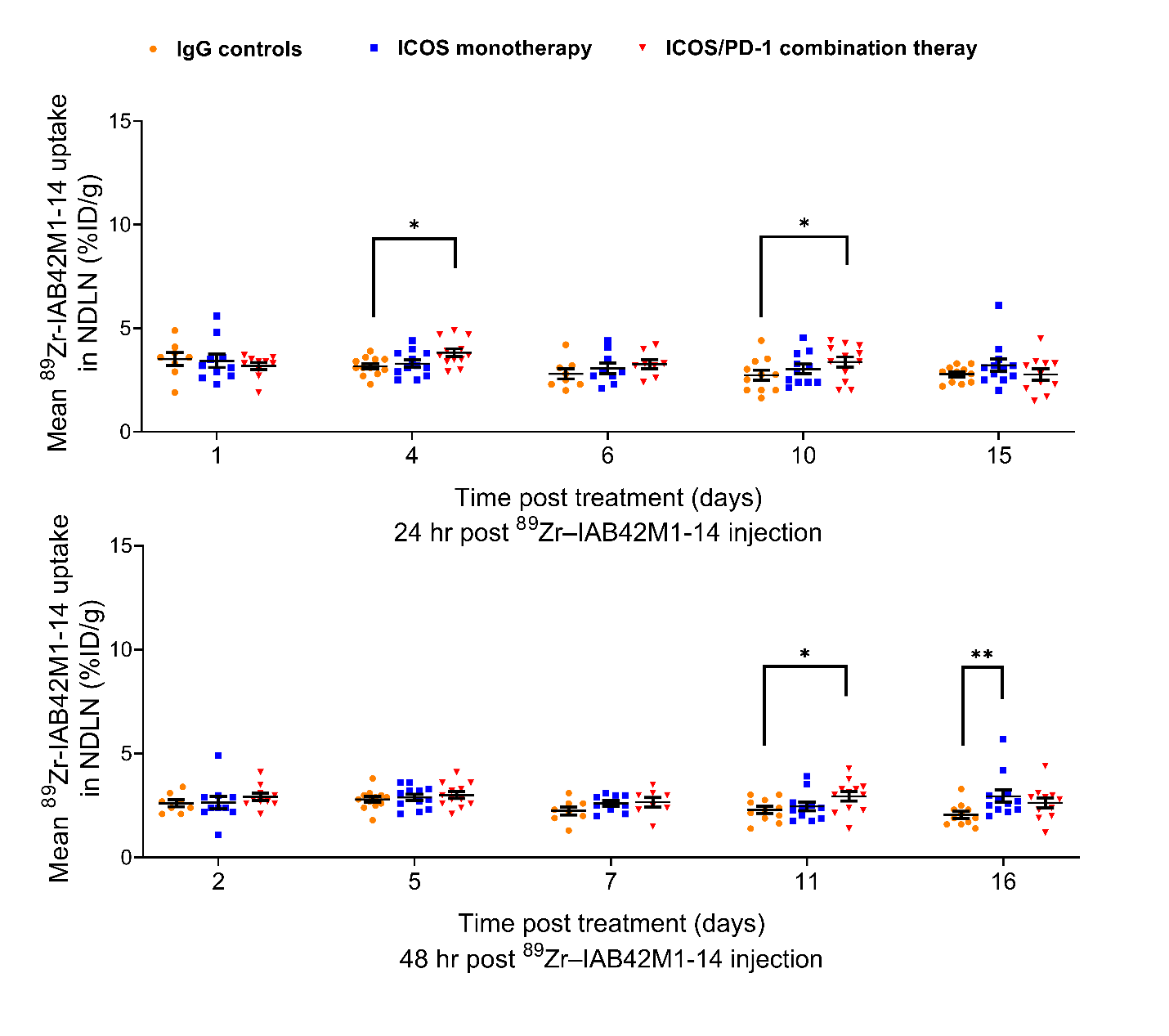
**Figure S2.** In-vivo ^89^Zr–IAB42M1-14 minibody uptake in the contralateral non-draining lymph node (NDLN) was significantly higher in the ICOS monotherapy group on day 16, and in the ICOS/PD-1 combination group on days 4, 10, and 11 compared to the IgG controls group. *P<0.05, **P<0.01 A linear mixed model with animal as a random effect was used and all pairwise group comparisons were performed within the same day).

**
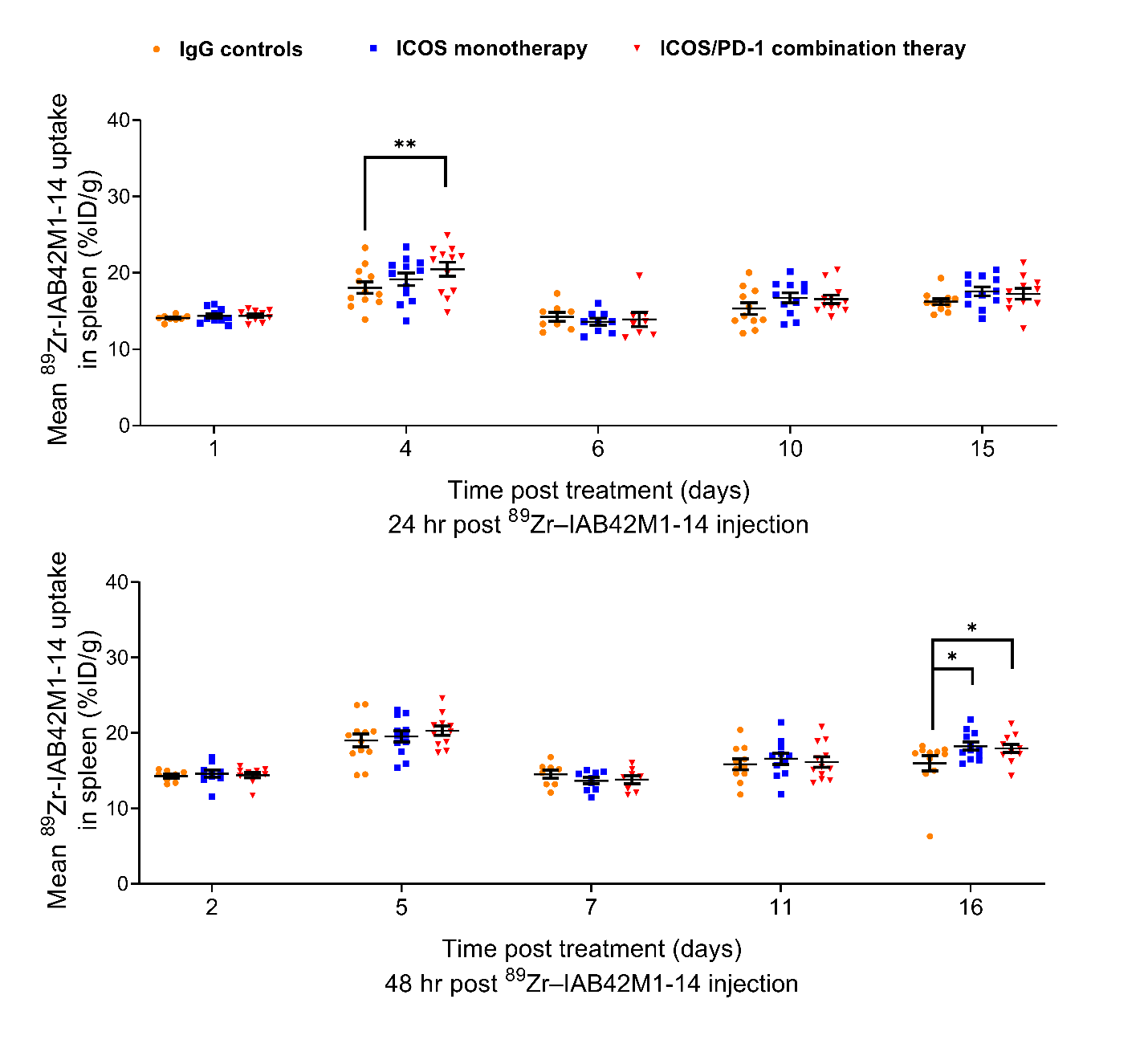
**

**Figure S3.** In-vivo ^89^Zr–IAB42M1-14 minibody uptake in the spleen was significantly higher in the ICOS monotherapy group on day 16, and in the ICOS/PD-1 combination group on days 4, and 16 compared to the IgG controls group. *P<0.05, **P<0.01. A linear mixed model with animal as a random effect was used and all pairwise group comparisons were performed within the same day).

**
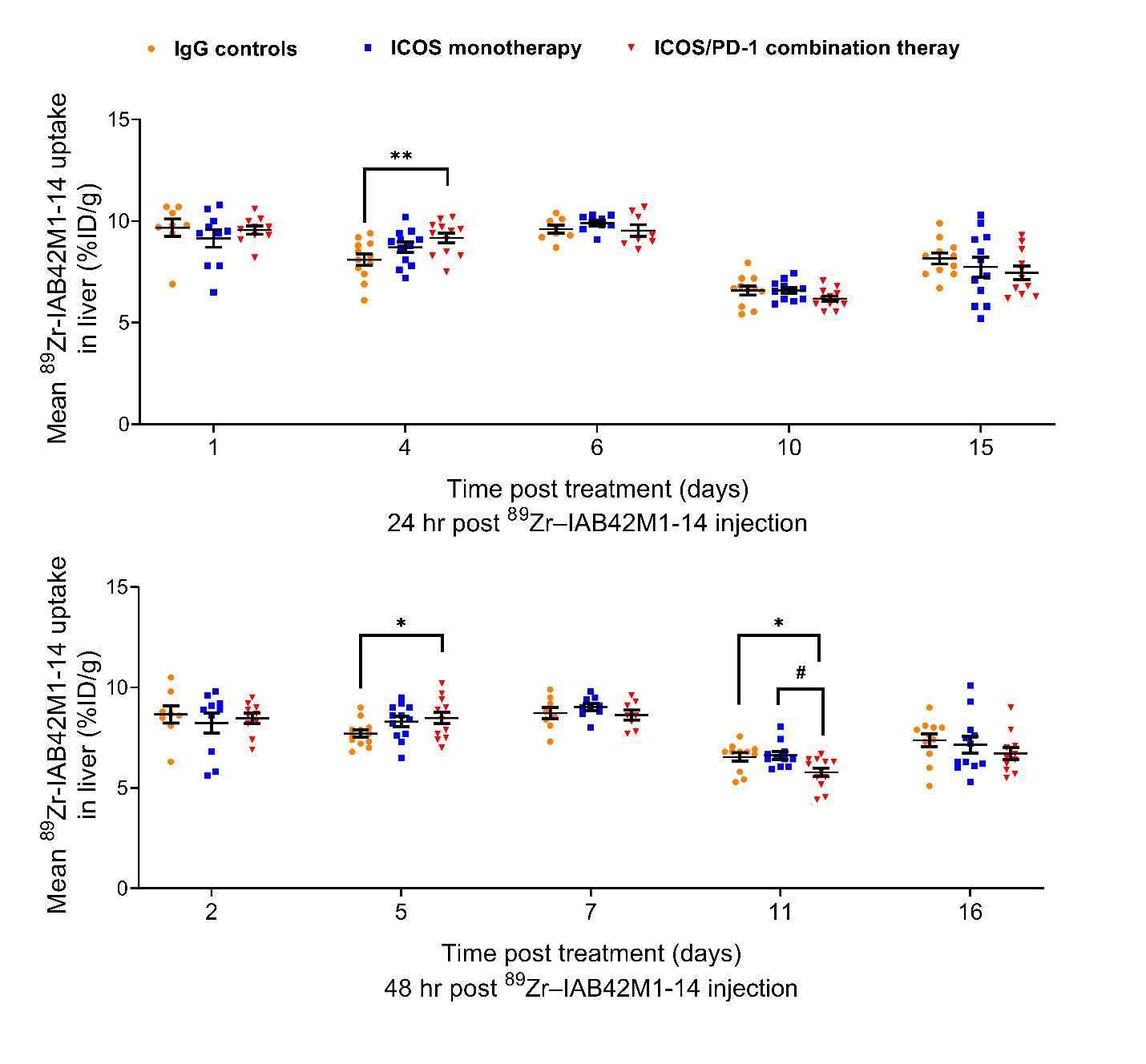
Figure S4.** In-vivo ^89^Zr– IAB42M1-14 minibody uptake in the liver was significantly different in the ICOS/PD-1 combination group on days 4, 5, and 11 compared to the IgG controls group (*P<0.05, **P<0.01), and in the ICOS monotherapy group on day 11 compared to the ICOS/PD-1 combination group (^#^P<0.05). A linear mixed model with animal as a random effect was used and all pairwise group comparisons were performed within the same day).

**
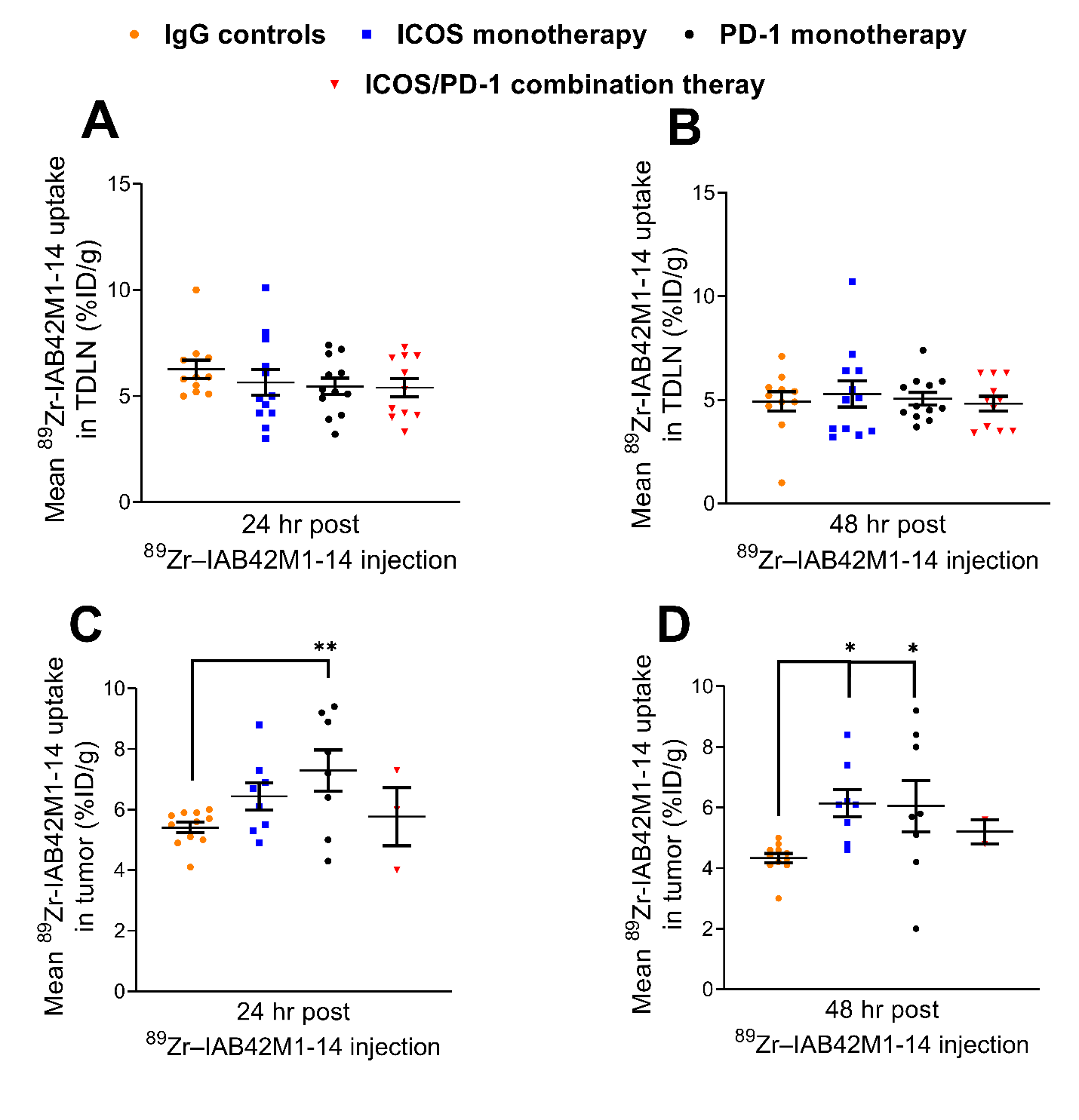
**

**Figure S5.**  Study 5 data, including PD-1 monotherapy.In-vivo ^89^Zr–IAB42M1-14 minibody uptake in TDLN **(A, B)** and in tumor **(C, D)** following treatment with ICOS or PD-1 monotherapies or ICOS/PD-1 combination, as measured using PET/CT imaging at 24 hr **(A, C)** or 48 hr **(B, D)** post minibody injection. No differences were observed in minibody uptake in TDLN in all treatment groups compared to IgG controls group. The uptake in the tumor was significantly higher in the PD-1 monotherapy group on Day 15, and 16, and in the ICOS monotherapy group on Day 16, compared to IgG controls group. *P<0.05, **P<0.01, ***P<0.001 A linear mixed model with animal as a random effect was used and all pairwise group comparisons were performed within the same day). Note that this analysis was conducted in addition to the primary analysis displayed in Fig. 5. that involved only three treatment groups; as a result, this analysis should be considered exploratory, and its findings of statistical significance should be interpreted accordingly.
